# Optimal low-intensity pulsed ultrasound stimulation for promoting anti-inflammatory effects in macrophages

**DOI:** 10.1063/5.0137881

**Published:** 2023-03-22

**Authors:** Francesco Iacoponi, Andrea Cafarelli, Francesco Fontana, Tiziano Pratellesi, Erik Dumont, Ivana Barravecchia, Debora Angeloni, Leonardo Ricotti

**Affiliations:** 1 The BioRobotics Institute, Scuola Superiore Sant'Anna, Piazza Martiri della Libertà 33, 56127 Pisa, Italy; 2 Department of Excellence in Robotics and AI, Scuola Superiore Sant'Anna, Piazza Martiri della Libertà 33, 56127 Pisa, Italy; 3 BAC Technology s.r.l., 50063 Florence, Italy; 4 Image Guided Therapy, 33600 Pessac, France; 5 Scuola Superiore Sant'Anna, Piazza Martiri della Libertà 33, 56127 Pisa, Italy

## Abstract

In this paper, we stimulated M1-like macrophages (obtained from U937 cells) with low-intensity pulsed ultrasound (LIPUS) to lower pro-inflammatory cytokine production. A systematic screening of different frequencies, intensities, duty cycles, and exposure times was performed. The optimal stimulation conditions leading to a marked decrease in the release of inflammatory cytokines were determined to be 38 kHz, 250 mW/cm^2^, 20%, and 90 min, respectively. Using these parameters, we verified that up to 72 h LIPUS did not affect cell viability, resulting in an increase in metabolic activity and in a reduction of reactive oxygen species (ROS) production. Moreover, we found that two mechanosensitive ion channels (PIEZO1 and TRPV1) were involved in the LIPUS-mediated cytokine release modulation. We also assessed the role of the nuclear factor κB (NF-κB) signaling pathway and observed an enhancement of actin polymerization. Finally, transcriptomic data suggested that the bioeffects of LIPUS treatment occur through the modulation of p38 MAPK signaling pathway.

## INTRODUCTION

Among all immune cells involved in the inflammatory process, macrophages represent the first line of defense against infections. They are located in all body tissues and arise from precursor cells, named monocytes. Tissue-resident macrophages and monocytes recruited from the bloodstream react in response to various inflammatory stimuli of the cellular microenvironment in which they reside or migrate, respectively.^1^ When inflammation is active, toll-like receptors (TLRs), especially TLR4, participate in the innate immune response causing the activation of signaling cascades in macrophages, such as nuclear factor kappa-light-chain-enhancer of activated B cells (NF-κB) and mitogen-activated protein kinase (MAPK),^2^ and the consequent production of high levels of inflammatory cytokines.^3^

Pro-inflammatory (M1-like) macrophages secrete a large variety of pro-inflammatory cytokines such as tumor necrosis factor-α (TNF-α), interleukin-1β (IL-1β), interleukin-6 (IL-6), and interleukin-8 (IL-8).^4,5^ Anti-inflammatory (M2-like) macrophages are able to secrete anti-inflammatory cytokines such as interleukin-4 (IL-4) and interleukin-10 (IL-10).^6^

The control of inflammation is a critical problem in the management of several diseases, such as cardiovascular pathologies, cancer, diabetes mellitus, and osteoarthritis.^7^ Currently, in the clinical setting, corticosteroids or anti-inflammatory drugs are commonly administered to reduce inflammation; however, all current pharmacological treatments are often far from being satisfactory, and may cause side effects, such as renal impairment, increased cardiovascular risk, and possible secondary infections.^8^ Low-intensity pulsed ultrasound (LIPUS) is a specific regime of ultrasound (US) stimulation, featured by frequencies > 20 kHz, low intensity (<3 W/cm^2^), and a pulsed waveform to minimize thermal effects. LIPUS is recently attracting more and more attention due to its ability to induce beneficial effects that promote tissue healing and regeneration.^9^

So far, the most promising results have been obtained by applying this technology to the field of fracture healing^10^ (LIPUS has been approved by the United States Food and Drug Administration for such an application^11^) and also for regeneration of soft tissues, such as cartilage, muscles, tendons, and ligaments.^12^ The possible mechanisms responsible for the above-mentioned effects on tissues and cells have been widely discussed, but they are still unclear and under continuous investigation.

The efficacy of LIPUS in regulating the inflammatory response linked to several diseases has also been recently explored.^13–15^ However, only a few groups have investigated the effect of LIPUS on macrophages. The most important results on this line of research are summarized in Table I. Feril *et al.*^16^ applied LIPUS to U937 cells, used as a leukemia model, demonstrating that apoptosis was maximized using a certain intensity, keeping all the other parameters fixed. Tabuchi *et al.*^17^ applied LIPUS on U937 with similar conditions, although with a fixed intensity value, and they observed a downregulation of 193 genes, including estrogen receptor 1 (ESR1), v-erb-b2 erythroblastic leukemia viral oncogene homolog 2 (ERBB2), and integrin β 1 (ITGB1), as well as an upregulation of 201 genes, including heme oxygenase (decycling) 1 (HMOX1), vimentin (VIM), and chemokine (C–C motif) ligand 3 (CCL3). In the work of Zhang *et al.*^18^ LIPUS was used with slightly different parameters, varying the intensity between 10 and 90 mW/cm^2^, and a decrement of the level of pro-inflammatory cytokines such as IL-1β, IL-6, and IL-8 in macrophage-like U937 cells was observed. The authors also observed an increase in cell viability, cell apoptosis inhibition, a suppression of degradation and phosphorylation of kappa-light-chain-enhancer of activated B cells (IκBα), and a translocation of NF-κB p65 subunit into nuclei. Other studies focused on LIPUS-induced bioeffects on the RAW 264.7 cell line, a murine model of macrophages, and on the THP-1 human cell line. In all these studies, the authors showed that LIPUS treatment, although performed with different conditions, inhibited the production of pro-inflammatory cytokines such as interleukin-33 (IL-33), IL-6, IL-8, and IL-1β and suppressed intracellular signaling such as extracellular signal-regulated kinase (ERK) and MAPK.^19–21^

**TABLE I.** Relevant *in vitro* studies focused on LIPUS stimulation of macrophages. Gene and protein symbols are in capital letters. ATP = adenosine triphosphate; CCL3 = chemokine (C-C motif) ligand 3; DC = duty cycle; ERBB2 = v-erb-b2 erythroblastic leukemia viral oncogene homolog 2; ESR1= estrogen receptor 1; ERK = extracellular signal-regulated kinase; F = frequency; HMOX1 = heme oxygenase (decycling) 1; I = intensity; IKBα = nuclear factor of kappa light polypeptide gene enhancer in B-cells inhibitor, alpha; ITGB1 = integrin beta 1; IL= interleukin; LIPUS = low-intensity pulsed ultrasound; LPS = lipopolysaccharide; MAPK = mitogen-activated protein kinase; PKM = pyruvate kinase muscle; PRF = pulse repetition frequency; SQSTM1 = sequestosome 1; t = exposure time; TNF = tumor necrosis factor; VIN = vimentin. N/A = not applicable.

Reference	Stimulation parameters	Cell line	Bio effects
16	F = 1 MHz; PRF = 0.1 kHz; I = 100–1000 mW/cm^2^; DC = 10 %; and t = 1 min.	U937	Optimal apoptosis with minimal lysis was attained 12 h after sonication at 300 mW/cm^2^.
17	F = 1 MHz; PRF = 0.1 kHz; I = 300 mW/cm^2^; DC = 10 %; and t = 1 min.	U937	Six hours after LIPUS treatment, apoptosis without cell lysis was observed. LIPUS downregulated 193 genes and upregulated 201 genes (associated with cellular movement and cell death).
18	F = 1.5 MHz; PRF = 1 kHz; I = 10, 30, 60, and 90 mW/cm^2^; DC = 20 %; and t = 2 h.	U937	LIPUS at 60 mW/cm^2^ was more effective in reducing IL-8 expression. LIPUS reduced the protein expression of IL-6 and IL-8 at both gene and protein levels.
			LIPUS primarily suppressed the degradation and phosphorylation of IKBα and the translocation of p65 into the nuclei.
19	F = 1.5 MHz; PRF = 0.25 kHz; I = 200 mW/cm^2^; DC = 20 %; and t = 20 min.	RAW 264.7	LIPUS was found to inhibit inflammation and decrease the levels of IL-1β, IL-33, IL-6, and IL-8.
20	F = 1 MHz; PRF = 0.1 kHz; I = 100 mW/cm^2^; DC = 20 %; and t = 20 min.	RAW 264.7	LIPUS treatment on RAW 264.7 inhibited the expression of pro-inflammatory cytokines (TNF-α and IL-6), activated caveolin-1, and suppressed p38 MAPK and ERK signaling.
21	F = 1.5 MHz; PRF = N/A; I = 30 mW/cm^2^; DC = 20 %; and t = 20 min.	THP-1 and RAW 264.7	LIPUS inhibited the production of IL-1β. In addition, LIPUS upregulated the autophagy level and accelerated the formation of an SQSTM1-PKM complex in the LPS-ATP-treated macrophages. In addition, LIPUS downregulated the level of PKM2 in LPS-ATP-treated macrophages.

However, in all the above-mentioned studies, a systematic screening of the different possible LIPUS parameters was not performed. Consequently, the optimal parameters producing anti-inflammatory effects are not known. Moreover, the LIPUS setups adopted did not guarantee precise control of the energy dose delivered to the target, thus, producing undesired acoustic artifacts, such as wave attenuations and reflections, which can hamper the repeatability of the experiments and can produce errors in the dose of energy delivered up to 700% with respect to the expected value.^22,23^

In this work, we investigated the anti-inflammatory bioeffects induced by LIPUS treatment on a human macrophage-like cell model by using a custom-made *in vitro* LIPUS stimulation system, with high control of the US dose transmitted to the cells.^24^ We assessed the effects of LIPUS on lipopolysaccharide (LPS)-induced U937 cells by exploring different frequencies (F: 38 kHz, 1 MHz, and 5 MHz), intensities (I: 25, 100, 250, and 450 mW/cm^2^), duty cycles (DC: 10%, 20%, 30%, and 40%) and stimulation times (t: 30, 60, 90, and 120 min) and measuring the release of pro-inflammatory cytokines (IL-1β, IL-8, and TNF-α). At the optimal LIPUS conditions, we also investigated more in-depth metabolic activity and ROS production. Moreover, we assessed the role of Piezo-Type Mechanosensitive Ion Channel Component 1 (PIEZO1), Transient Receptor Potential Cation Channel Subfamily V Member 1 (TRPV1), NF-κB signaling pathway, actin polymerization pathway, and modulation of gene expression pathways downstream of MAPK in the LIPUS-triggered cell response.

## RESULTS

### Phenotypic differentiation of U937 induced by PMA and selection of LPS concentration

As shown in Fig. S1(a), the exposure to PMA for 48 h led U937 mononuclear monocyte-like cells to differentiate into a M0 phenotype. The cells showed a flat morphology and were attached to the polystyrene (PS) surface. Moreover, as shown in Fig. S1(b), an increase in CD14 gene expression for all PMA-stimulated samples compared to the control sample (U937 without PMA) was observed. M0 cells were further skewed toward an M1-like phenotype by increasing concentrations of LPS for 24 h. The phenotype was confirmed by the increase in CD80 and CD86 markers (both characteristic of M1-like macrophages). As shown in Fig. S1(b), there was a statistical difference in the expression level of TNF-α between control and LPS experimental groups when the LPS concentration was above 1 *μ*g/ml. In addition, no morphological changes were observed in M1-like macrophages induced with 1 *μ*g/ml LPS. Thus, this concentration was used in all the subsequent experiments.

### Optimization of stimulation with US

**FIG. 1.**
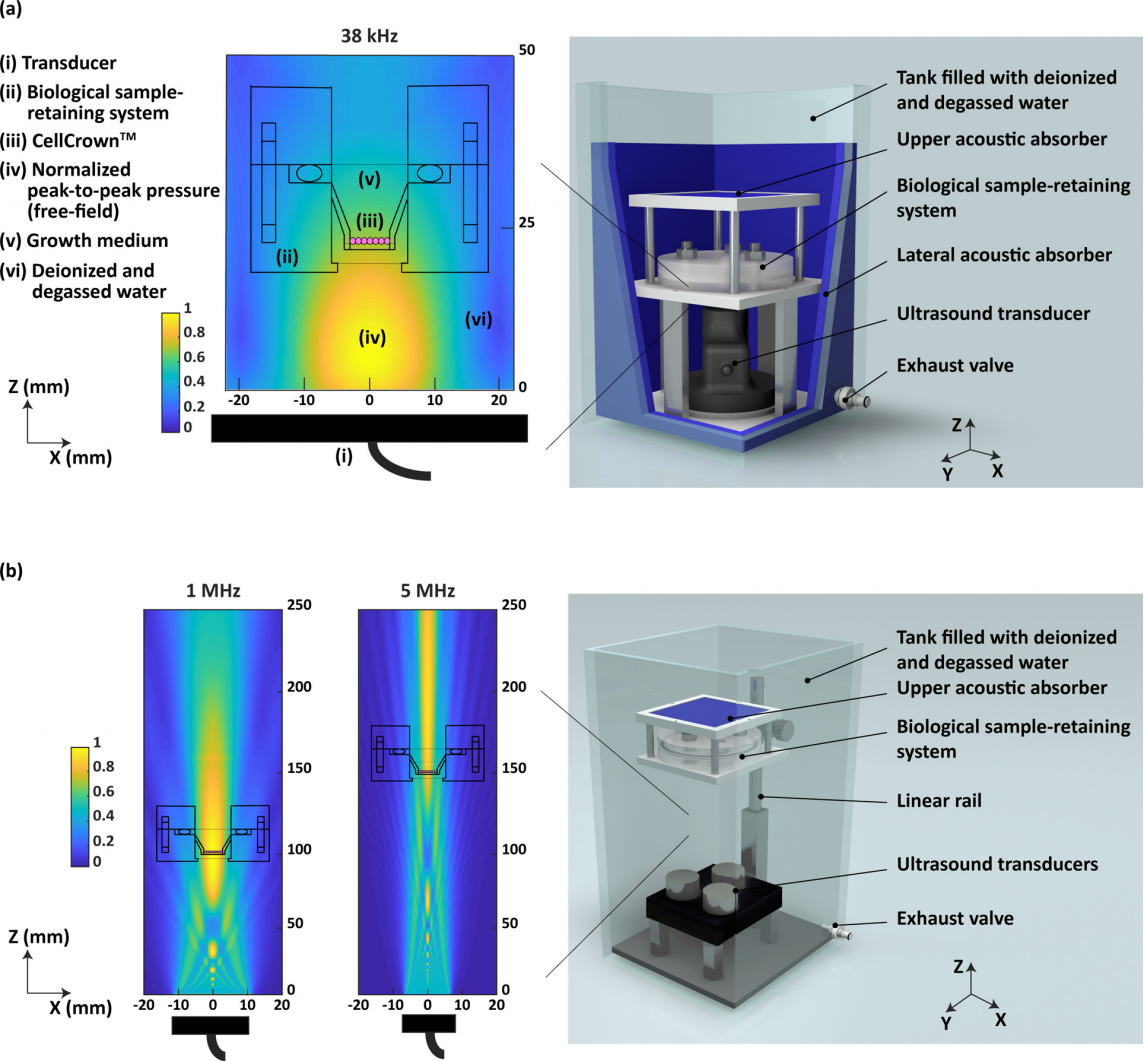
Stimulation setups adopted in the study. (a) Depiction of the low-F stimulation system with a schematic of the normalized peak-to-peak pressure field reaching the CellCrown^TM^ at 38 kHz and (b) depiction of the high-F stimulation system with a schematic of the normalized peak-to-peak pressure field hitting the CellCrown^TM^ at 1 and 5 MHz.

Two custom-designed LIPUS setups,^25^ dedicated to low and high F, respectively, were adopted in this work to perform highly controlled LIPUS stimulations. The low-F system [Fig. 1(a)] allowed to stimulate biological samples at 38 kHz. The high-F system [Fig. 1(b)] allowed to perform stimulations at 1 MHz and 5 MHz, by also exploring different I, DC, and t values.

**FIG. 2.**
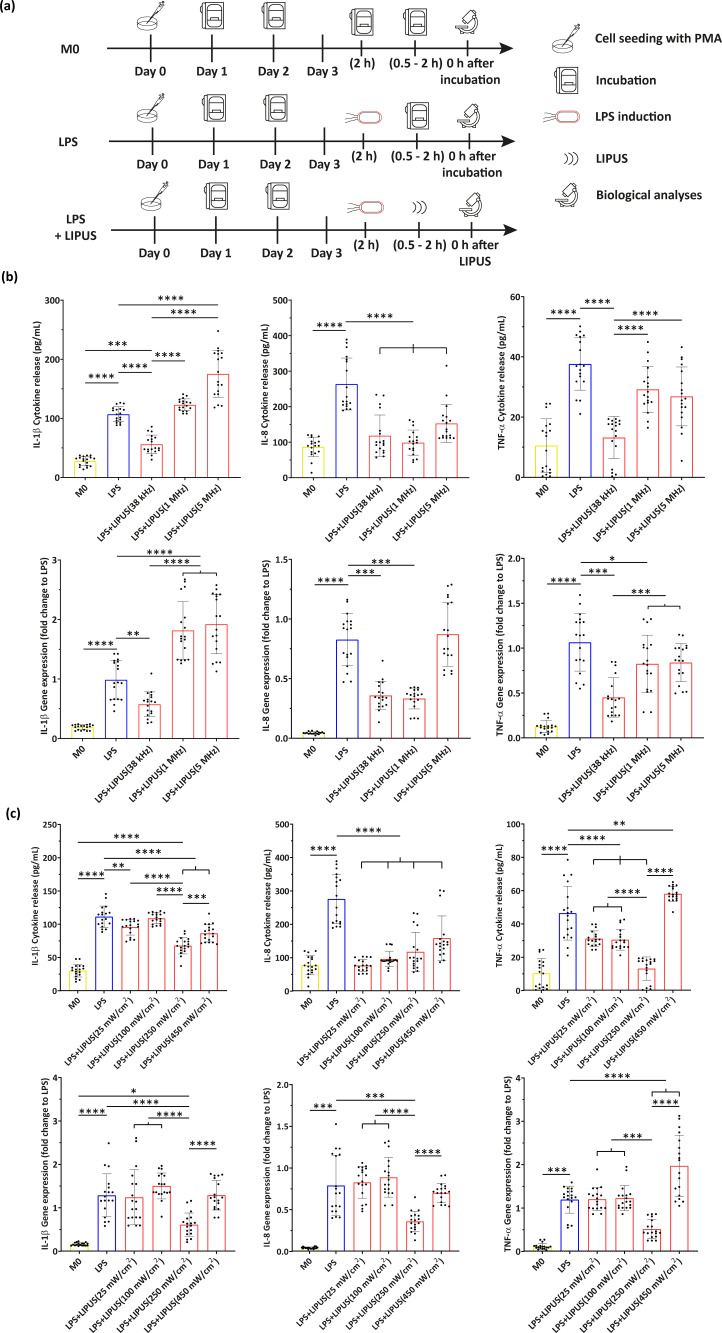
Results of the LIPUS parameter optimization. (a) Experimental groups related to the optimization of US parameters with the relative timeline; (b) evaluation of IL-1β, IL-8, and TNF-α with F variation (38 kHz, 1 MHz, and 5 MHz), in terms of cytokine release and gene expression. Only statistical differences with respect to LPS and “LPS+LIPUS(38 kHz)” groups are depicted. (c) Evaluation of IL-1β, IL-8, and TNF-α with I variation (25, 100, 250, and 450 mW/cm^2^), in terms of cytokine release and gene expression. Only statistical differences with respect to LPS and “LPS+LIPUS(250 mW/cm^2^)” groups are depicted. ^*^ = p < 0.05; ^**^ = p < 0.01; ^***^ = p < 0.001; and ^****^ = p < 0.0001. N = 9.

By means of the above-mentioned systems, different LIPUS conditions were explored, taking into account three experimental groups [Fig. 2(a)]: in M0, cells were not treated with LPS or LIPUS (i.e., negative control); in LPS, cells were treated with LPS for 2 h but they were not treated with LIPUS (i.e., positive control); in LPS+LIPUS, cells were treated with LPS for 2 h and then they were subjected to LIPUS stimulation.

At first, three values of F were explored: 38 kHz, 1 MHz, and 5 MHz. In this experiment, I, DC, and t were kept fixed at 250 mW/cm^2^, 20%, and 120 min, respectively (chosen as quite broadly used values in the literature). The results are shown in Fig. 2(b). It can be observed that LPS treatment increased the level of all the three cytokines (i.e., TNF-α, IL-1β, and IL-8) with respect to the M0 group. With regard to the exploration of different F, 38 kHz considerably decreased the level of all the analyzed pro-inflammatory cytokines, both at protein and gene expression levels, thus, resulting in more effectiveness than other stimulation conditions. Differently, 5 MHz was never able to downregulate IL-1β, IL-8, and TNF-α production both at protein and gene level, compared to the LPS group; a F of 1 MHz, instead, was able to lower only IL-8 production at a protein level. Therefore, 38 kHz was selected and fixed for the following screenings.

Then, a similar protocol was used, but exploring four values of I: 25, 100, 250, and 450 mW/cm^2^ (F was set at the optimal value of 38 kHz found in the previous experiment, whereas DC and t were set at 20% and 120 min, respectively). The results are shown in Fig. 2(c).

Concerning the definition of optimal I, 250 mW/cm^2^ was found to significantly downregulate cytokines release with respect to other I values. The I of 450 mW/cm^2^ had no remarkable effect, whereas 25 and 100 mW/cm^2^ were able to lower only IL-8 production at the protein level. Therefore, the I of 250 mW/cm^2^ was selected for further analyses.

**FIG. 3.**
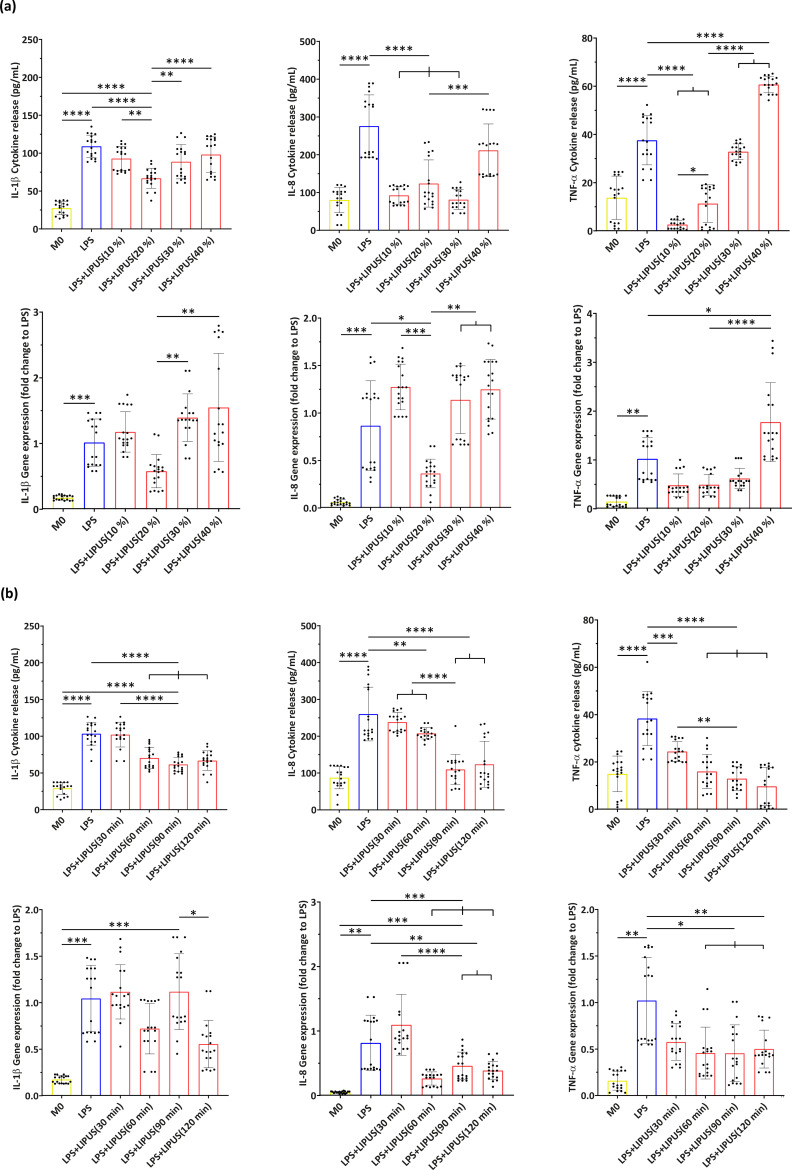
Results of the LIPUS parameter optimization. (a) Evaluation of IL-1β, IL-8, and TNF-α with DC variation (10%, 20%, 30%, and 40%), in terms of cytokine release and gene expression. Only statistical differences with respect to LPS and “LPS+LIPUS(20%)” groups are depicted. (b) Evaluation of IL-1β, IL-8, and TNF-α at the four explored t (30, 60, 90, and 120 min), in terms of cytokine release and gene expression. Only statistical differences with respect to LPS and “LPS+LIPUS(90 min)” groups are depicted. ^*^ = p < 0.05; ^**^ = p < 0.01; ^***^ = p < 0.001; and ^****^ = p < 0.0001. N = 9.

With F fixed at 38 kHz and I fixed at 250 mW/cm^2^, the role of DC was explored, considering 10%, 20%, 30%, and 40% values. The results are shown in Fig. 3(a). A DC of 20% resulted in the most effective lowering of the inflammatory level, with respect to the other conditions; 10% was also effective but only on IL-8 and TNF-α protein releases, whereas 30% lowered only the IL-8 protein release. So, together with a F of 38 kHz and an I of 250 mW/cm^2^, a DC of 20% was chosen among the selected parameters for the following investigation, where t was varied between 30, 60, 90, and 120 min. The results are shown in Fig. 3(b). We found that the treatment efficacy reached a plateau behavior starting from a t of 90 min, so this value was chosen as the minimum optimal t.

Overall, the following combination of parameters was found to be the most effective in decreasing pro-inflammatory cytokine production: F = 38 kHz; I = 250 mW/cm^2^; DC = 20%, and t = 90 min.

### Effects on cell viability, metabolism, and intracellular ROS

**FIG. 4.**
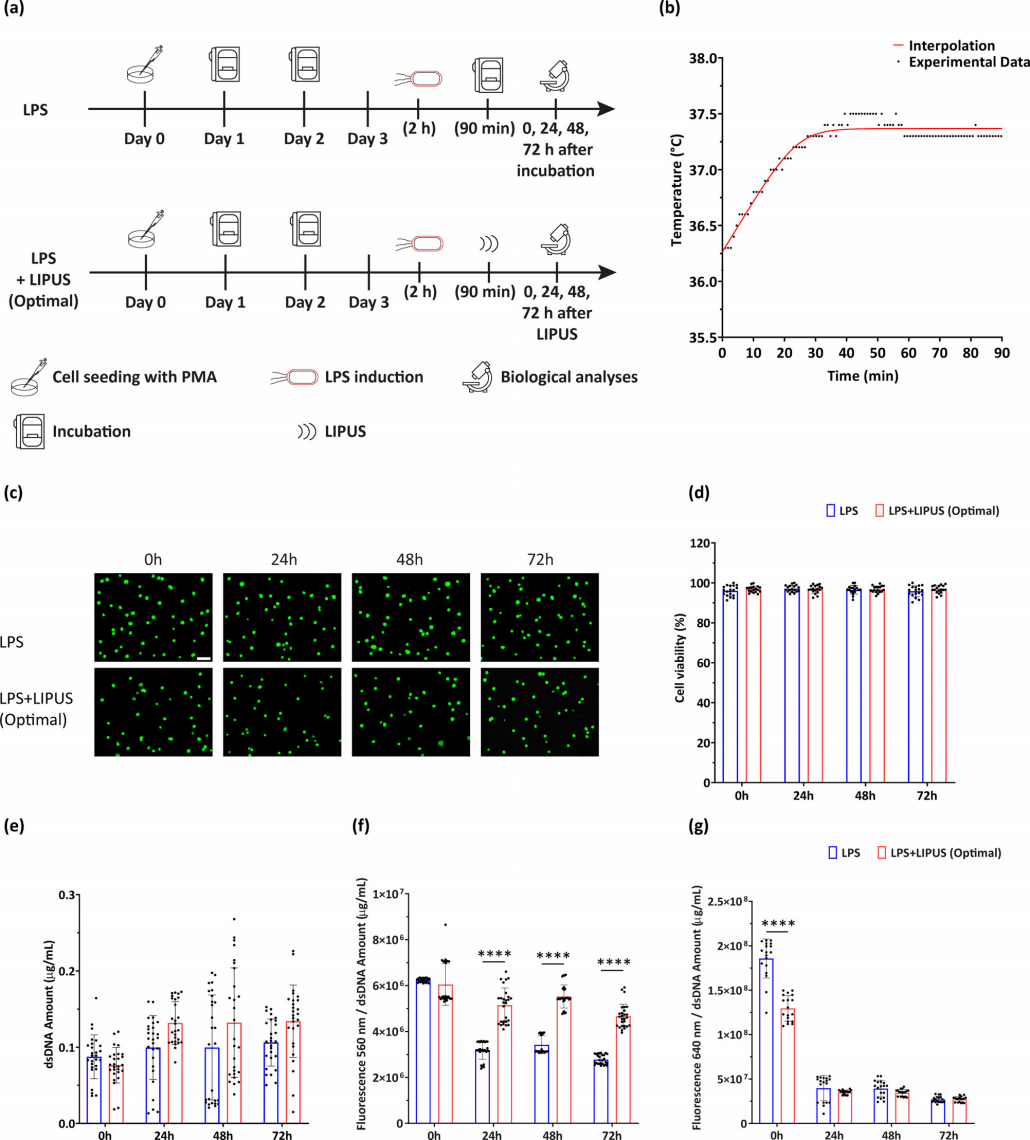
Temperature monitoring and observation of effect on viability, metabolism, and ROS release. (a) Experimental groups related to viability, metabolism, and ROS evaluation with the relative timeline (LIPUS stimulation protocol: F = 38 kHz, I = 250 mW/cm^2^, DC = 20%, and t = 90 min). (b) Measurements of temperature variation over time. (c) Representative LIVE/DEAD images (green: viable cells; red: necrotic or dead cells; scale bar: 100 *μ*m) and (d) quantitative cell viability assessment of LPS and LPS+LIPUS (Optimal) groups at multiple time points post-stimulation. N = 9. (e) Evaluation of cell proliferation, (f) metabolic activity, and (g) intracellular ROS at multiple time points post-stimulation. Metabolic activity and ROS production were expressed in terms of fluorescence signal (A.U.) over the dsDNA amount (*μ*g/ml). ^****^ = p < 0.0001. A.U. = arbitrary units. N = 9.

Two experimental groups were defined [Fig. 4(a)], named (i) LPS, where M0 cells were treated with LPS for 2 h but were not treated with LIPUS (i.e., positive control), and (ii) LPS+LIPUS (Optimal), where M0 cells were treated with LPS for 2 h and then stimulated at the optimal stimulation conditions for all the US parameters (i.e., F = 38 kHz, I = 250 mW/cm^2^, DC = 20%, and t = 90 min; PRF =1 kHz).

As shown in Fig. 4(b), the maximum temperature increase recorded during LIPUS stimulation was around 1 °C, which is in line with previous reports.^26,27^ Therefore, the treatment can be classified as non-thermal.

Concerning cell viability, representative images of LPS and “LPS+LIPUS (Optimal)” groups are shown in Fig. 4(c). No significant differences were observed between control and stimulated samples, up to 72 h post-stimulation. These qualitative results were in agreement with dsDNA analyses at the same time point [Fig. 4(d)], in which no statistically significant differences were observed between all the conditions.

The results of metabolic activity and ROS production are reported in Figs. 4(e) and 4(f), respectively. The outcome of both these tests was normalized with respect to dsDNA amount. An increase in cell metabolic activity was clearly observed in the LPS+LIPUS (Optimal) group with respect to the LPS one, for all the time points.

Concerning ROS, no statistical difference was found between LPS and LPS+LIPUS (Optimal) groups from 24 to 72 h post-stimulation. However, LIPUS at the optimal stimulation condition was able to considerably reduce ROS production immediately after stimulation.

### Temporal evolution of pro-inflammatory cytokines release

**FIG. 5.**
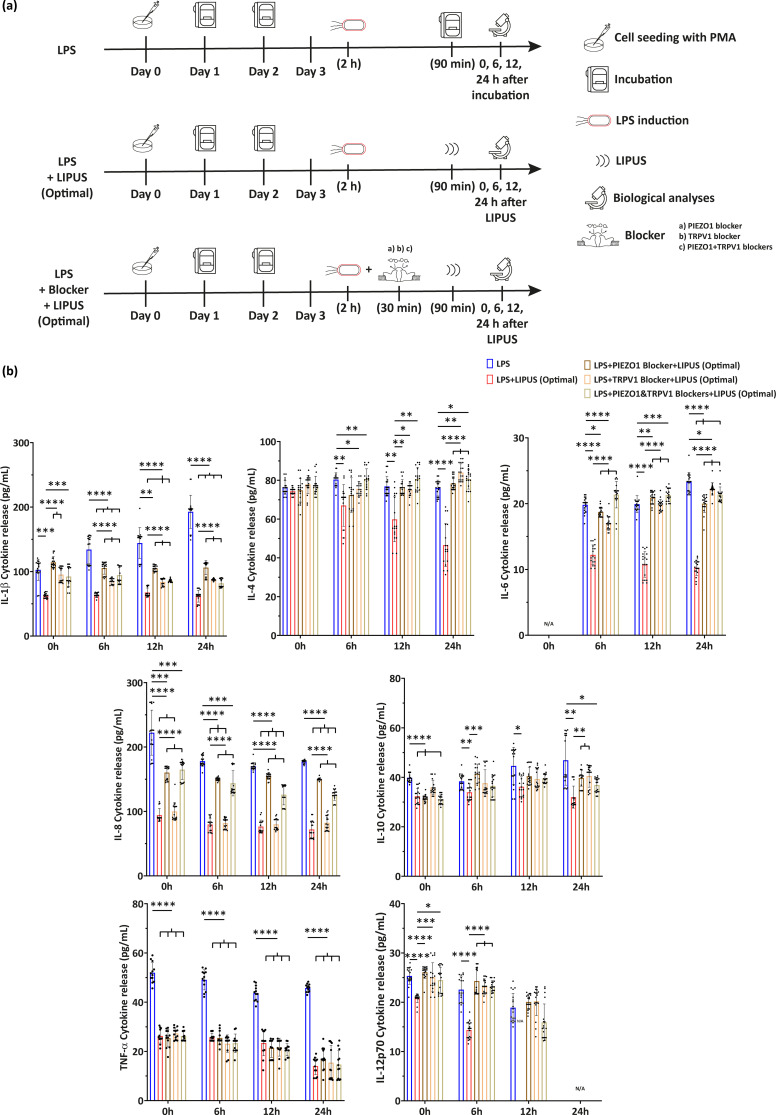
Cytokine release at multiple timepoints. (a) Experimental groups related to multiple timepoint pro-inflammatory cytokine release in the presence of selective ion channel blockers, with the relative timeline. (b) Evaluation of IL-1β, IL-4, IL-6, IL-8, IL-10, TNF-α, and IL-12p70 cytokine release at the optimal LIPUS stimulation conditions (F = 38 kHz, I = 250 mW/cm^2^, DC = 20%, and t = 90 min) in the presence or absence of selective mechanoresponsive ion channel blockers. ^*^ = p < 0.05; ^**^ = p < 0.01; ^***^ = p < 0.001; and ^****^ = p < 0.0001. N/A = not applicable. N = 9.

Five experimental groups were defined [Fig. 5(a)], named as follows: (i) LPS, where M0 cells were treated with LPS for 2 h but not treated with LIPUS; (ii) LPS+LIPUS (Optimal), where M0 cells were treated with LPS for 2 h and then stimulated with LIPUS at the optimal stimulation conditions found during US parameter screening (i.e., F = 38 kHz, I = 250 mW/cm^2^, DC = 20%, and t = 90 min); (iii) LPS+Blocker+LIPUS groups, where M0 cells were treated with LPS, then treated with selective ion channel blockers [with (iii) PIEZO1 blocker, (iv) BCTC blocker or (v) both of them] and then stimulated at the optimal stimulation conditions found during US parameter screening (i.e., F = 38 kHz, I = 250 mW/cm^2^, DC = 20%, t = 90 min, and PRF = 1 kHz).

**FIG. 6.**
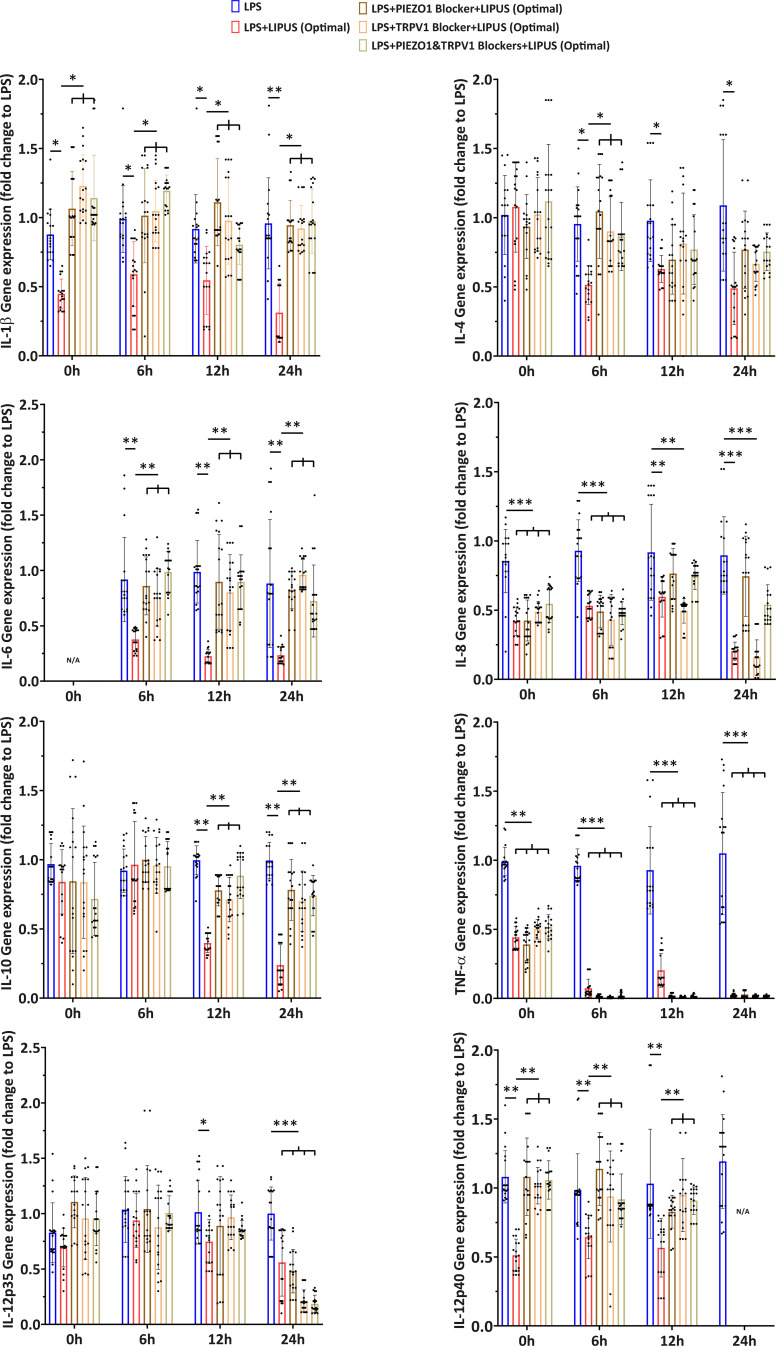
Gene expression at multiple timepoints. Evaluation of IL-1β, IL-4, IL-6, IL-8, IL-10, TNF-α, IL-12p35, and IL-12p40 gene expression at optimal LIPUS stimulation conditions (F = 38 kHz, I = 250 mW/cm^2^, DC = 20%, and t = 90 min) in the presence or absence of selective mechanoresponsive ion channel blockers. Data of the gene expression were calculated as fold change with respect to the LPS group. ^*^ = p < 0.05; ^**^ = p < 0.01; ^***^ = p < 0.001; and ^****^ = p < 0.0001. N/A = not applicable. N = 9.

Multiple cytokine production by LPS-induced M0 cells over 72 h post LIPUS treatment at the optimal stimulation conditions were analyzed. The results are shown in Figs. 5 and 6. IL-6 production appeared relatively late, in line with previous studies on human monocytes,^28^ while an increase in IL-6-encoding mRNA levels was detected 6 h post-stimulation. For IL-6, both at protein and gene expression levels, the LPS+LIPUS (Optimal) group was statistically lower than the LPS group and the three “LPS+Blocker+LIPUS (Optimal)” groups.

Concerning IL-1β, the behavior was the same as that of IL-6, both at protein and gene expression levels: the LPS+LIPUS (Optimal) group was significantly different from the LPS group for all the time points, and the three LPS+Blocker+LIPUS (Optimal) groups were statistically different from the LPS+LIPUS (Optimal) group for all time points.

As regards IL-8, the LPS+LIPUS (Optimal) group and the “LPS+TRPV1 Blocker+LIPUS (Optimal)” group showed lower values than the other experimental groups, for all considered time points, at protein level. Interestingly, at gene expression level, also the “LPS+PIEZO1 Blocker+LIPUS (Optimal)” group and the “LPS+PIEZO1&TRPV1 Blocker+LIPUS (Optimal)” group were significantly different from the LPS group, up to 6 h post-stimulation. From 12 h post-stimulation, the trend at gene level followed the same kinetics of the protein release.

Regarding TNF-α, all experimental groups were significantly different from the LPS group, at each time point, both at protein and gene levels. For IL-12p35 gene expression, the “LPS+LIPUS” group was significantly different from the LPS group starting from 12 h after stimulation, whereas 24 h post-stimulation, all the LIPUS-stimulated groups were statistically lower with respect to the LPS group. Interestingly, the IL-12p40 subunit exhibited different kinetics, in which the LPS+LIPUS (Optimal) group remained at lower levels than the other groups, up to 12 h post-stimulation. At 24-h time point, only the gene expression of the LPS group was detectable.

Regarding IL-12p70 cytokine production, similar kinetics as IL-12p40 were observed up to 6 h post-stimulation. At the 12-h timepoint, the trend was also identical, with the difference that no protein release was detected in the LPS+LIPUS (Optimal) group. After 24 h, only the LPS group was detected: this latter result was likely due to the additive effect of the anti-inflammatory LIPUS stimulation and the upregulation of IL-4, which, according to the literature, was able to independently suppress IL-12p70 in *in vitro* monocyte-derived macrophages.^29^ Regarding IL-4, a decrease over time was observed in the LPS+LIPUS (Optimal) group at both protein and gene levels; gene expression of IL-10, on the other hand, decreased from 12 h post-stimulation.

### Inhibition of NF-κBp65 and modification of actin organization

**FIG. 7.**
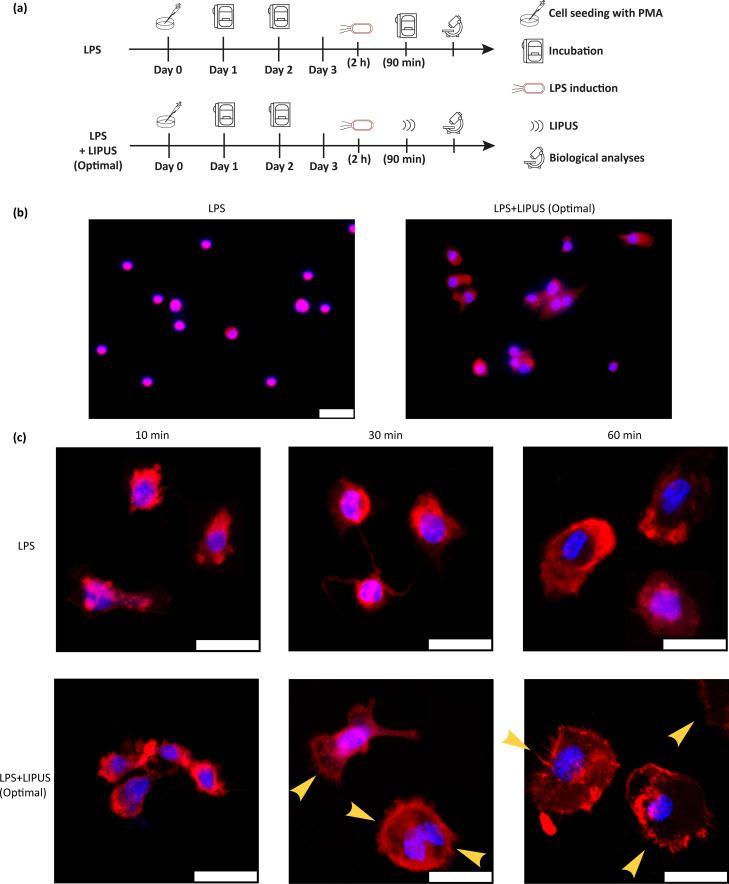
Observation of NF-κBp65 and actin polymerization. (a) Experimental groups related to NF-κBp65, actin polymerization, and MAPK pathways analysis, with the relative timeline. (b) Representative immunofluorescence images of LPS and LPS+LIPUS (Optimal) groups, in which the inhibition of nuclear translocation of p65 can be observed. Red: p65, blue: nuclei. Scale bar: 25 *μ*m. N = 9. (c) Representative fluorescence images (maximum intensity projections) of LPS and LPS+LIPUS (Optimal) groups, 10 (left column), 30 (center column), and 60 (right column) minutes after LIPUS stimulation. The US treatment induced a marked increase in actin polymerization, visible from 30 min post-stimulation. Red: F-actin, blue: nuclei. Scale bar: 25 *μ*m. Yellow arrows indicate the formed microspikes. N = 9.

Two experimental groups were defined [Fig. 7(a)], named (i) LPS, where M0 cells were treated with LPS for 2 h but were not treated with LIPUS (i.e., positive control), and (ii) LPS+LIPUS (Optimal), where M0 cells were treated with LPS for 2 h and then stimulated at the optimal stimulation conditions found during US parameter screening (i.e., F = 38 kHz, I = 250 mW/cm^2^, DC = 20%, t = 90 min, and PRF = 1 kHz).

The LPS treatment induced the nuclear translocation of the p65 subunit. LIPUS treatment significantly reversed this process, as clearly visible in Fig. 7(b); on the left, a purple nuclear halo is visible, resulting from the superposition of blue (chromatin) and red (p65) signals; on the right, the red signal is cytoplasmic. Moreover, the formation of microspikes was evident 30 min after the stimulation [Fig. 7(c)], suggesting that the LIPUS treatment was capable of rearranging the actin cytoskeleton and appeared as a trigger capable of accelerating phagocytosis.

### LIPUS induce the overexpression of genes involved in the p38 MAPK Pathway

To determine whether the LIPUS stimulation could affect the MAPK pathway, we performed a transcriptome analysis comparing the LPS group with the LPS+LIPUS(Optimal) group. We used RT2 profiler PCR arrays that analyzed 83 specific genes involved in the MAPK pathway. MAPK cascades are key signaling pathways that regulate a broad variety of cellular processes, including proliferation, differentiation, apoptosis, and stress responses. The MAPK pathway includes three main kinases: MAPK kinase kinase, MAPK kinase, and MAPK, which activate and phosphorylate downstream proteins.^30^ The MAPKs in mammals include c-Jun NH2-terminal kinase (JNK), p38 MAPK, and extracellular signal-regulated kinase (ERK). Studies have shown that the JNK and p38 MAPK pathways are mainly related to stress (oxidative, genotoxic, and osmotic stress as well as by proinflammatory cytokines) and apoptosis of cells, while the ERK/MAPK signaling pathway, which is one of the most studied, is closely related to cell proliferation and differentiation.^31^ We found an upregulation of 17 out of 83 genes, while none was downregulated by the treatment (see Table II and Fig. S4). Therefore, the transcriptomic analysis suggested a general activation of p38 MAPK pathways (genes such as TP53, MAPK9, MAPK14, and MAPK12 were all upregulated).

**TABLE II.** Transcriptome analysis related to MAPK pathway. Deregulated genes in LPS+LIPUS(Optimal) samples, compared to “LPS” samples with a fold change at least of ±2 and a p-value less than 0.05 are listed. The geometric mean of two housekeeping/reference genes (B2M and RPL0) was used to normalize the raw data. The p-values were calculated based on a Student's t-test of the replicate 2̂ (−ΔCT) values for each gene in the control group (LPS) and treatment group (LPS+LIPUS). The p-value calculation used is based on parametric, unpaired, two-sample equal variance, and two-tailed distribution. ATF2 = Activating transcription factor 2; CCND2 = Cyclin D2; CREBBP = CREB binding protein; ELK1 = ELK1, member of ETS oncogene family; FOS = FBJ murine osteosarcoma viral oncogene homolog; HRAS = V-Ha-ras Harvey rat sarcoma viral oncogene homolog; HSPB1 = heat shock 27 kDa protein 1; LAMTOR3 = late endosomal/lysosomal adaptor, MAPK and MTOR activator 3; MAP2K7 = mitogen-activated protein kinase kinase 7; MAP3K1 = mitogen-activated protein kinase kinase kinase 1; MAPK12 = mitogen-activated protein kinase 12; MAPK13 = mitogen-activated protein kinase 13; MAPK14 = mitogen-activated protein kinase 14; MAPK8IP2= mitogen-activated protein kinase 8 interacting protein; MAPK9 = mitogen-activated protein kinase 9; MAPKAPK2 = mitogen-activated protein kinase-activated protein kinase 2; TP53 = tumor protein p53.

Gene name	Fold change	p-value
ATF2	2.23	0.0075
CCND2	2.73	0.0129
CREBBP	4.02	0.0223
ELK1	11.22	0.0236
FOS	14.39	0.0140
HRAS	3.13	0.0322
HSPB1	7.32	0.0365
LAMTOR3	3.19	0.0107
MAP2K7	3.21	0.0299
MAP3K1	2.45	0.0225
MAPK12	3.38	0.0206
MAPK13	3.81	0.0416
MAPK14	3.23	0.0477
MAPK8IP2	3.22	0.0119
MAPK9	2.57	0.0469
MAPKAPK2	2.93	0.0264
TP53	4.65	0.0097

This is an interesting result since the p38 MAPKs pathway plays an important role in the cascades of cellular responses evoked by a wide range of external signals (such as mechanical stress) and responds appropriately by generating a plethora of different biological effects leading to direct activation of transcription factors.^32–38^

## DISCUSSION

Macrophages have a fundamental role in immune response and inflammation.^39^ Better control of the inflammatory response remains, at the moment, an open and unsolved line of research, to face several pathologies. Our results showed that M0 macrophages expressed CD14, confirming that PMA activated U937 efficiently, as already shown previously.^40,41^ LPS-stimulated macrophages expressed CD80 and CD86 markers and TNF-α starting from 1 μg/mL of the compound, which therefore was chosen as the most appropriate concentration, also in agreement with previous studies.^18,42^

Our results clarified what are the optimal LIPUS parameters triggering anti-inflammatory effects on human macrophages (U937 cell line) activated with a strong pro-inflammatory compound such as LPS. No previous studies systematically screened different US parameters (F, I, DC, and t), which may lead to different responses at a cellular level, to identify the optimal protocol for inflammation reduction *in vitro*. Due to its minimal side effects and non-invasiveness,^9^ LIPUS is used as a treatment for lowering intracellular inflammatory factors. Zhang *et al.*^18^ observed (at F = 1.5 MHz, I = 60 mW/cm^2^, DC = 20%, and t = 2 h) a downregulation of apoptotic rate and pro-inflammatory cytokines and increased cell viability, compared to unstimulated control, but their stimulation protocol was not focused on properly controlling the US dose: in fact, all the US parameters were fixed except for the I, for which four different values were tested and their relative bioeffects investigated. However, the poor control of the US dose at the target hampers the reproducibility of the experiments and a full understanding of the interaction between the stimulus and the induced biological effects, thus, slowing down the possible clinical translation.

To assess the optimal parameters able to lower the inflammatory level induced by LPS, ELISA analysis and real-time qRT-PCR analysis of three key pro-inflammatory cytokines released by macrophages (TNF-α, IL-1β, and IL-8) were performed. US stimulation at 38 kHz was found to be the best F tested, considerably lowering the inflammatory level of all the three cytokines tested, both at protein and gene levels, compared to 1 and 5 MHz stimulation. This result represented an absolute novelty in the state of the art and, in general, it is the first attempt to explore such low F level, since, as reported by Abrunhosa *et al.*,^43^ US is typically used in the 0.5–5 MHz range. The I and DC ranges tested in this study are the typical ones adopted in LIPUS stimulation state of the art, which do not cause significant thermal effects.^9^ Concerning I, although 25 and 100 mW/cm^2^ triggered positive effects related to cytokines release, a I of 250 mW/cm^2^ is able to lower the inflammatory level more. When considering DC, 20% guaranteed a greater lowering of the LPS-induced inflammatory level. The exploration of multiple t, as well as multiple DC, was also never explored in the state of the art: in this study, a 90-min stimulation corresponded to the minimal optimal time that guaranteed a significant lowering of proinflammatory cytokines. The results derived from this parameter optimization are of considerable importance because they could be used as a guideline for future pre-clinical and clinical protocols of anti-inflammatory therapies based on LIPUS therapy.

Remarkably, additional tests confirmed that this specific stimulation protocol resulted also in an improvement in terms of cell metabolism and intracellular ROS production suppression, without affecting cell viability. Cell proliferation remained at a steady state since PMA induction, which caused cell cycle arrest before the differentiation step into M0 macrophages.^44^

Ion channels are porous membrane proteins, necessary to modify membrane potentials and to tune action potentials and other electrical signals by controlling the passage of ions.^45^ They are involved in the modulation of molecular immune cell environment by acting on inflammatory or anti-inflammatory cascades.^46^ Early findings have pointed out the key role of ion channels in immune cell behaviors.^47^ PIEZO family of genes were discovered in 2010 as potential channels in the cell line Neuro2A, a glial tumor line.^48^ Among PIEZO channels, PIEZO1 plays a key role in a variety of cell activities, such as cell differentiation, homeostasis,^49^ and red blood cell regulation.^50^ Moreover, PIEZO1 is the most expressed mechanosensitive ion channel in macrophages and an important regulator in macrophage mechanosensing response.^51^ TRP channels are a family of ion channels located on the plasma membrane. TRPV1 is a member of the TRP channel family, responsible for nociceptive, thermal, and mechanical sensations; it has been shown that its expression is present in many cell types including immune cells, so it plays a pivotal role in inflammation and immunity processes.^52^ By using specific channel blockers, we demonstrated for the first time the key role of these PIEZO1 and TRPV1 ion channels on macrophages when LIPUS is applied.

For some cytokines, both in the case of protein release in the supernatant and gene expression, the inflammatory level of the LPS+Blocker+LIPUS (Optimal) groups was considerably higher than the one of LPS+LIPUS (Optimal) group: this could suggest that the opening of the channel, when not blocked, promoted the activation of the anti-inflammatory signaling cascade and the consequent lowering of the inflammatory level. For example, IL-1β, IL-6, and TNF-α production, both at protein and gene expression levels, seemed to be directly affected by these ion channels at all the investigating timepoints; also IL-8 production, at gene expression level, seemed to be affected when PIEZO1 (or both PIEZO1 and TRPV1) was applied. Moreover, the treatment efficacy, when optimal stimulation parameters were applied, was confirmed in terms of pro-inflammatory and anti-inflammatory cytokine release modification, at multiple timepoints, both analyzing the protein release in the supernatant and gene expression. It is worth noting that the level of IL-10, a notorious anti-inflammatory cytokine, did not increase, as would be expected in a generalized anti-inflammatory context, and, in fact, it decreased upon LIPUS. However, it is possible that the anti-inflammatory conditions applied in this *in vitro* model were not sufficient to overcome LPS effect, widely recognized as a potent inflammatory activator in macrophages.^53^

NF-κB is a family of transcription factors, including RelA (p65), RelB, c-Rel, NF-κB1 (p105), and NF-κB2 (p100).^54^ It controls cytokines transcription and antimicrobial effectors as well as genes that regulate cell differentiation, proliferation, and survival.^55^ It was demonstrated that, *in vitro*, NF-κB is activated by LPS,^56^ and NF-κBp65 contributes to an inflammatory response in macrophages by regulating the expression of various genes such as TNF-α, IL-1β, IL-6, and IL-8.^57^ It has been reported that LIPUS inhibited NF-κB nuclear translocation in U937 cells.^18^ The study of Chen *et al.*^58^ also reported that LIPUS attenuated LPS-induced neuroinflammation by modulating TLR4/NF-κB pathway. Our results showed that LPS treatment forced p65 to enter the nuclei, whereas, on the other hand, LIPUS stimulation inhibited p65 protein expression and NF-κB nuclear translocation, thereby suggesting that the anti-inflammatory effect of LIPUS occurs at least in part through the NF-κB pathway.

Monocytes and resident tissue macrophages are key regulators of critical biological processes such as tissue repair and regeneration.^59^ Active monocytes/macrophages require actin cytoskeletal remodeling for chemotaxis and phagocytosis that produces distinct F-actin-rich membrane structures.^60–64^ Ultrasound stimulation reportedly triggers cellular mechanotransduction machinery, such as phosphorylation of focal adhesion complex, disturbed retrograde of actin cytoskeleton, and abnormal membrane protrusion, as observed in different cell types under different exposure conditions.^65–68^ Zhou *et al.*^69^ observed that LIPUS stimulation accelerated phagocytosis in macrophages via a mechanism that enhances actin polymerization. This result was also similar to a previous observation on primary skin fibroblasts, published by the same research group.^70^ The study of Carballo *et al.*^71^ reported that PMA and LPS treatment induces a transformation of actin structure in terms of microspike disappearance, with respect to the non-treated control. Our work shows that LIPUS stimulation, in our specific experimental settings, possibly through the activation of PIEZO1 and TRPV1, although on a background of LPS-activated macrophages, did not cause damage on microspike formation, as microspikes and lamellipodia are again visible already 30 min after stimulation in treated cells. This observation reinforces the hypothesis that LIPUS can revert the inflammatory phenotype to a more basal situation of “primed” macrophage that shows the dynamic formation of microspikes and lamellipodia.

In summary, in this work, we stimulated LPS-induced M0 macrophages through a highly controlled US system, analyzing several stimulation parameters (F, I, DC, and t) to optimize the treatment and maximize the desired bioeffect, namely, inflammatory cytokine release reduction. We demonstrated that a F of 38 kHz, a I of 250 mW/cm^2^, a DC of 20%, and a t of 90 min lower the inflammatory environment up to 24 h post-stimulation, do not affect the cell viability, increase cell metabolism, changed actin polymerization, and inhibited ROS production and NF-κB activation. Noticeably, by blocking mechanoresponsive membrane ion channels such as PIEZO1 and TRPV1, LIPUS stimulation did not lower the inflammatory level of cytokines in some experimental groups, suggesting that the aforementioned channels are primarily responsible for the interaction between mechanical wave and the subsequent inhibition of inflammatory pathways in the cell. The analysis of gene expression pathways modified by LIPUS in LPS-treated macrophages showed that 17 genes, among over 90 tested, were activated. They all belong to p38 pathway and, based on the literature, are involved in mechanosensing.^32–36^ It must be noted that not all genes tested changed their expression level, showing that LIPUS has a certain specificity of action on them. The observation that some pro-inflammatory cytokines decreased after LIPUS suggested that those 17 genes might play a major role in the anti-inflammatory activity of LIPUS. Further studies are necessary to characterize this phenomenon more in detail, for example, by investigating the cross-interaction with multiple membrane channels, also at different time points. Future efforts should also aim to minimize the exposure time (looking for a compromise between anti-inflammatory effects and time needed for the stimulation), thus to ease a possible future clinical translation of LIPUS-mediated inflammation treatments.

## METHODS

### Architecture of the LIPUS setups

Two custom-designed LIPUS setups described in Fontana *et al.*,^25^ dedicated to low and high F, respectively, were adopted in this work to perform highly controlled LIPUS stimulations. The low-F system [Fig. 1(a)] allowed stimulating biological samples at 38 kHz by adopting a 50 mm-diameter piezoelectric flat transducer (BAC s.r.l., Florence, Italy), whose acoustic field was previously characterized in free field conditions.^25^ The transducer was electrically activated by a 2W maximum power signal generator (SIRIO, BAC s.r.l., Florence, Italy), with the possibility to vary I, DC, pulsed repetition frequency (PRF), and t. For the high-F range (1–5 MHz), another LIPUS setup was used, allowing the stimulation of three biological samples at the same time. Three piezoceramic transducers of the same type (23 mm diameter—1 MHz central F for stimulating samples at 1 MHz; 15 mm diameter—4 MHz central F for stimulating samples at 5 MHz) (Precision Acoustics, Dorchester, Dorset, UK) were also previously characterized in terms of pressure field maps and I calibration [Fig. 1(b)]. The transducers were powered by a 4-channels (2 W/channel) signal generator (Image Guided Therapy, Pessac, France) with dedicated software for setting I, DC, PRF, and t. Both for the low- and high-F setups, biological samples were hosted in a biological sample-retaining system (BSRS)^72^ during US stimulations. The BSRS was designed to prevent undesired acoustic reflections and attenuations through the use of thin membranes (38 *μ*m thick polyurethane film, Stretchlon200^®^, Airtech International Inc.) mounted in correspondence with the acoustic path. Also, the BSRS ensured biological sample sterility through a chamber that was sealed to external contaminations, since it was immersed in deionized and degassed water during the stimulation session. Biological samples were seeded on a 29 *μ*m PS film mounted within a CellCrown^TM^24NX insert (Scaffdex, Finland). Such insert could be easily transferred from multiwell plates (kept in incubator) to the BSRS for the duration of the LIPUS stimulation, and vice versa.

### Cell line and culture conditions

Human mononuclear monocytes-like U937 cells (ATCC, CRL-1593.2), a model widely used in the state of the art to investigate a variety of biological processes related to monocyte and macrophage functions, were maintained in growth medium (GM) composed of RPMI-1640 medium (Corning, cat. no. 10–040-CV) supplemented with 10% v/v fetal bovine serum (FBS, Sigma-Aldrich, cat. no. F0804) and 1% v/v penicillin/streptomycin (P/S, Sigma-Aldrich, cat. no. P4333) in a 5% carbon dioxide humidified atmosphere at 37 °C. Approximately, 100 000 U937/ml were seeded on a PS film (Goodfellow, cat. no. 126–603-64) mounted within a CellCrown^TM^ insert (Sigma-Aldrich, cat. no. Z742381), allowing for ease of transfer to the stimulation system for the duration of treatment. Before cell seeding, CellCrown^TM^ inserts were treated with oxygen plasma (Tucano Plasma RF 13.56 MHz, Gambetti. Parameters: 100% O_2_, 50 s @ 50 W, and 0.6 mbar) to improve the hydrophilicity of the PS substrate. According to the canonical classification, M0 macrophages are defined as undifferentiated macrophages with the potential to polarize into specific macrophage subtypes.^73^ Therefore, cells started the differentiation process into an adherent, non-polarized macrophage phenotype M0 by adding 50 ng/ml phorbol 12-myristate 13-acetate (PMA, Sigma-Aldrich, cat. no. P8139) to GM, in a 5% carbon dioxide humidified atmosphere, at 37 °C, for 48 h. Cells were then washed with 1X phosphate-buffered saline (PBS, Sigma-Aldrich, cat. no. D1283) to remove the extra PMA, and fresh GM was added for the next experiments. LPS is the major component of Gram-negative bacteria cell walls, and it is widely recognized as a potent activator of monocytes/macrophages, causing inflammatory response by triggering the release of a large plethora of inflammatory cytokines.^74^ To model an appropriate inflammatory response during LIPUS experiments, M0 were treated with LPS (from *Escherichia coli* O111:B4, Sigma-Aldrich, cat. no. L2630) at 0.1, 0.5, 1, 5, and 10 *μ*g/ml for 24 h, in order to find the best LPS concentration to induce inflammation. In particular, the minimum LPS concentration that induced a statistical difference in TNF-α release with respect to the negative control (M0 not treated with LPS) was considered sufficient to induce an appropriate inflammation state in the M0 macrophages.

#### Cytokine release quantification

Cell supernatant was collected, and cytokine production was analyzed with Human TNF-α ELISA kit (Invitrogen, cat. no. KHC3011), Human IL-1β ELISA kit (Invitrogen, cat. no. BMS224–2), and Human IL-8 ELISA kit (Invitrogen, cat. no. BMS204–3), following the manufacturer's instructions. A VICTOR Nivo Multilabel plate reader (PerkinElmer, Waltham, MA, USA) was used to read the absorbance signal, setting a primary wavelength of 450 nm for all the kits. The results were converted to numeric values using standard curves.

#### Gene expression analysis

Total RNA was isolated with 500 *μ*l TRIzol^®^ reagent (Invitrogen, cat. no. 15596018) and extracted with the RNeasy Micro kit (QIAGEN, cat. no. 74004), following the manufacturer's instructions for processing adherent cells. RNA was quantified with a Nanodrop™ 2000 (Thermo Scientific, Waltham, MA, USA). Reverse transcription (RT) and real-time qRT-PCR was performed with KAPA SYBR^®^ FAST One-Step qRT-PCR Master Mix (2X) Universal kit (Sigma-Aldrich, cat. no. KK4651) in a Rotor-Gene® Q System (QIAGEN, Germantown, MD, USA) according to the manufacturer's instructions. Forward and reverse primers for real-time qRT-PCR amplification of TNF-α, IL-1β, and IL-8 are listed in Table SI. The relative gene expressions were normalized to glyceraldehyde-3-phosphate dehydrogenase (GAPDH), analyzed with the ΔΔC(T) method^75^ and expressed as fold change, equivalent to the ratio of the target group over the control group (M0).

### Temperature measurements

Temperature increase induced in biological samples at the optimal stimulation conditions was measured with a fine-wire type T thermocouple (cat. no. KA01, T. M. Electronics, resolution of 0.1 °C) positioned inside the BSRS. Temperature data were acquired at a sampling F of 1 Hz with a thermocouple measurement device (USB-TC01, National Instruments) that was connected to a PC.

### Effects on cell viability, metabolism, and intracellular ROS

#### Viability assay

Cell cultures were analyzed using a LIVE/DEAD® Viability/Cytotoxicity assay (Invitrogen, cat. no. L3224). Briefly, GM was removed and replaced with 1X PBS containing 2 *μ*M calcein-AM and 4 *μ*M ethidium homodimer-1 (EthD-1). After incubation at room temperature for 30 min, cells were washed with 1X PBS and observed under a Leica DMi8 microscope (Leica Microsystems, Wetzlar, Germany).

#### Cell proliferation assay

Cell cultures were analyzed using a Quant-iT™ PicoGreen™ dsDNA Assay Kit (Invitrogen, cat. no. p11496). Briefly, cells were lysed in 500 *μ*l of nuclease-free water (Sigma-Aldrich, cat. no. W4502); aliquots of 50 *μ*l were transferred in a 96-well black round bottom PS microplate (Corning, cat. no. 3792), prepared according to the manufacturer's instructions. After 10 min of incubation in the dark at room temperature, fluorescence intensity was read with VICTOR Nivo Multilabel plate reader, setting an excitation wavelength of 485 nm and an emission wavelength of 535 nm. The results were converted to numeric values using standard curves.

#### Metabolic activity assay

Cell cultures were analyzed using a PrestoBlue™ Cell Viability Reagent (Invitrogen, cat. no. A13262). Briefly, GM was removed and changed to RPMI-1640 containing 10% v/v of the reagent described above. After incubating the solution in a 96-well black round bottom PS microplate at 37 °C for 60 min, VICTOR Nivo Multilabel plate reader was used to read the fluorescence signal, setting an excitation wavelength of 560 nm and an emission wavelength of 590 nm.

#### Intracellular ROS assay

Cell cultures were analyzed using a Fluorometric Intracellular ROS kit (Sigma-Aldrich, cat. no. MAK142). ROS fluorescence was detected using ROS Detection Reagent according to the manufacturer's protocols. After incubating the solution in a 96-well black round bottom PS microplate at 37 °C for 60 min, VICTOR Nivo Multilabel plate reader was used to read the fluorescence signal, setting an excitation wavelength of 640 nm and an emission wavelength of 675 nm.

### Multiple timepoint sampling of pro-inflammatory cytokines

#### Inhibition of ion channels

GsMTx-4 (Abcam, cat. no. ab141871) and BCTC (Abcam, cat. no. ab141517) were used as selective blockers of PIEZO1 channel and TRPV1 channel, respectively. GsMTx-4 (3 *μ*M) and BCTC (10 *μ*M) were added to the medium 30 min before LIPUS treatment.

#### Cytokine release quantification

Cell supernatant was collected, and cytokine production was analyzed with Human TNF-α ELISA kit (Invitrogen, cat. no. KHC3011), Human IL-1β ELISA kit (Invitrogen, cat. no. BMS224–2), Human IL-4 ELISA kit (Invitrogen, cat. no. BMS225–2), Human IL-6 ELISA kit (Invitrogen, cat. no. BMS213–2), Human IL-8 ELISA kit (Invitrogen, cat. no. BMS204–3), Human IL-10 ELISA kit (Sigma-Aldrich, cat. no. RAB0244), and Human IL-12p70 (Invitrogen, cat. no. KHC1578), following the manufacturer's instructions. A VICTOR Nivo Multilabel plate reader was used to read the absorbance signal, setting a primary wavelength of 450 nm for all the kits. The results were converted to numeric values using standard curves.

#### Gene expression analysis

Total RNA was isolated with 500 μl TRIzol^®^ reagent (Invitrogen, cat. no. 15596018) and extracted with the RNeasy Micro kit (QIAGEN, cat. no. 74004), following the manufacturer's instructions for processing adherent cells. RNA was quantified with a Nanodrop™ 2000 (Thermo Scientific, Waltham, MA, USA). Reverse transcription and real-time qRT-PCR were performed with KAPA SYBR^®^ FAST One-Step qRT-PCR Master Mix (2X) Universal kit (Sigma-Aldrich, cat. no. KK4651) in a Rotor-Gene® Q System (QIAGEN, Germantown, MD, USA), according to the manufacturer's instructions. Forward and reverse primers for real-time qRT-PCR amplification of CD14, CD80, CD86, TNF-α, IL-1β, IL-4, IL-6, IL-8, IL-10, IL-12p35, IL-12p40, and NF-κB p65 are listed in Table SI. The relative gene expressions were normalized to GAPDH, analyzed with the ΔΔC(T) method^75^ and expressed as fold change, equivalent to the ratio of the target group over the control group (LPS).

### Inhibition of NF-κBp65, enhancement of actin polymerization, and activation of MAPK pathway

#### Immunofluorescence staining evaluation

Cells were fixed with 4% paraformaldehyde (PFA, ThermoFisher, cat. no. 28908) in 1X PBS for 10 min, and then, permeated with 0.1% Triton X-100 (Sigma-Aldrich, cat. no. T8787) in 1X PBS for 15 min and blocked with 5% bovine serum albumin (BSA, PAN-Biotech, cat. no. P06–139310) in 1X PBS-0.1% TWEEN20 (Sigma-Aldrich, cat. no. P2287) solution for 30 min. Samples were incubated with a primary antibody against NF-κBp65 (1:200, Abcam, cat. no. ab1652) overnight, followed by a secondary antibody (1:500, Invitrogen, cat. no. a11036) and Hoechst 33342 (1:1000, Invitrogen, cat. no. H3570) for 1 h in the dark. After three washes with 1X PBS, slides were observed under a Leica DMi8 microscope.

#### F-Actin and nuclear staining evaluation

Cells were fixed with 4% PFA in 1X PBS for 10 min, then permeabilized with 0.1% Triton X-100 in 1X PBS for 15 min and blocked with 5% BSA in 1X PBS-0.1% TWEEN20 solution for 30 min. Samples were incubated with Phalloidin-TRITC (1:1000, Sigma-Aldrich, cat. no. P1951) and Hoechst 33342 (1:1000) for 1 h in the dark. After three washes with 1X PBS, a CLSM system with NISElements software (Nikon, Amsterdam, Netherlands) was used to acquire two-dimensional confocal images.

#### RT2 profiler PCR arrays

Transcriptome analysis was performed with RT2 Profiler PCR Arrays (96-well format) for Human MAP Kinase Signaling Pathway (QIAGEN, cat. no. 330231). A quantity of 50 ng total RNA was used to generate cDNA using the RT2 First Strand Kit; cDNA pre-amplification was obtained by RT2 PreAMP cDNA Synthesis Kit (QIAGEN, cat. no. 330451) and RT2 PreAMP Pathway Primer Mixes (QIAGEN, cat. no. 330241) using a multiplex, PCR-based preamplification, according to the manufacturer's instructions, to provide amplification of gene-specific cDNA target templates. Real-time qRT-PCR was performed with CFX96 Touch Real-Time PCR Detection System (BioRad, Hercules, CA, USA). Data analysis was carried out with QIAGEN'S Gene Globe Data Analysis Center using a software-based tool. The data analysis web portal calculates fold change/regulation using ΔΔC(T) method.^75^ Fold change was then calculated using 2̂ (-ΔΔCT) formula and has been set at ± 2. Only up- or down-regulated genes with a p-value < 0.05 were reported.

### Statistical analyses

The experiments were independently performed three times, and three independent biological samples were used for each experiment. Data showed a normal distribution (Shapiro–Wilk normality test, p = 0.05), so statistical analysis was performed using parametric tests. The results were expressed as mean ± standard deviation and graphically shown as bar plots. One-way ANOVA with Tukey's multiple comparisons test (GraphPad Prism 8, GraphPad Software Inc.) was used to verify significant differences in the optimization of US parameter experiments. Student t-test was used to assess differences in the trascriptome analysis, whereas in all the other experiments two-way ANOVA with Sidak's multiple comparison test was used to assess differences between groups. The results were considered statistically different for p < 0.05. Statistical differences were defined as ^*^p < 0.05, ^**^p < 0.01, ^***^p < 0.001, and ^****^p < 0.0001.

## SUPPLEMENTARY MATERIAL

See the supplementary material for details on the phenotypic differentiation of U937 cells, on the overall results of the LIPUS parameter optimization, on the scatter plot and clustergram of the transcriptome analysis and on the oligonucleotide primer sequences.

## Data Availability

The data that support the findings of this study are available from the corresponding author upon reasonable request.
